# Chemosensory gene expression in olfactory organs of the anthropophilic *Anopheles coluzzii* and zoophilic *Anopheles quadriannulatus*

**DOI:** 10.1186/s12864-017-4122-7

**Published:** 2017-09-22

**Authors:** G. Athrey, L. V. Cosme, Z. Popkin-Hall, S. Pathikonda, W. Takken, M. A. Slotman

**Affiliations:** 1 Department of Poultry Science, Texas A&M University, College Station, TX USA; 2 Department of Ecology and Evolutionary Biology, Yale University, New Haven, CT USA; 3 Department of Entomology, Texas A&M University, College Station, TX 77845 USA; 4 Laboratory of Entomology, Wageningen University, Wageningen, The Netherlands

**Keywords:** Olfaction, Chemosensory genes, Host seeking, Malaria vector, RNAseq, Antennae, Maxillary palp

## Abstract

**Background:**

*Anopheles (An.) coluzzii,* one of Africa’s primary malaria vectors, is highly anthropophilic. This human host preference contributes greatly to its ability to transmit malaria. In contrast, the closely related *An. quadriannulatus* prefers to feed on bovids and is not thought to contribute to malaria transmission. The diverged preference for host odor profiles between these sibling species is likely reflected in chemosensory gene expression levels in the olfactory organs. Therefore, we compared the transcriptomes of the antennae and maxillary palps between *An. coluzzii* and *An. quadriannulatus*, focusing on the major chemosensory gene families.

**Results:**

While chemosensory gene expression is strongly correlated between the two species, various chemosensory genes show significantly enhanced expression in one of the species. In the antennae of *An. coluzzii* the expression of six olfactory receptors (*Ors)* and seven ionotropic receptors (*Irs*) is considerably enhanced, whereas 11 *Ors* and 3 *Irs* are upregulated in *An. quadriannulatus*. In the maxillary palps, leaving aside *Irs* with very low level of expression, one *Ir* is strongly enhanced in each species. In addition, we find divergence in odorant binding protein (*Obp*) gene expression, with several highly expressed *Obps* being enhanced in the antennae and palps of *An. coluzzii.* Finally, the expression of several gustatory receptors (*Grs*) in the palps appears to be species-specific, including a homolog of a sugar-sensing *Drosophila Gr.*

**Conclusions:**

A considerable number of *Ors* and *Irs* are differentially expressed between these two closely related species with diverging host preference. These chemosensory genes could play a role in the human host preference of the malaria vector *An. coluzzii*. Additionally, divergence in *Obp* expression between the two species suggests a possible role of these odor carrier proteins in determining host preference. Finally, divergence in chemosensory expression in the palps may point towards a possible role for the maxillary palps in host differentiation.

**Electronic supplementary material:**

The online version of this article (10.1186/s12864-017-4122-7) contains supplementary material, which is available to authorized users.

## Background

Several mosquitoes of the *Anopheles gambiae* complex are among the most important vectors of human malaria in sub-Saharan Africa. A major determinant for the effectiveness of these species as malaria vectors is their degree of human hosts preference. The species in the complex vary considerably in their host preference, with the highly anthropophilic *An. gambiae s.s.* and *An. coluzzii,* collectively referred to as *An. gambiae s.l.* hereafter, at one end of the spectrum, and the zoophilic *An. quadriannulatus*, which rarely feeds on humans, at the other [1]. The high preference of *An. gambiae* s.l. for human hosts translates into a high vectorial capacity for human malaria. In contrast, *An. quadriannulatus,* a competent vector, albeit less so than *An. gambiae* s.l. [2, 3], is not thought to contribute to malaria transmission because it rarely feeds on humans in the field [1], although it readily does so in the lab [4].

Host seeking in mosquitoes is primarily modulated by the olfactory system. The antennae and the maxillary palps are the two main olfactory appendages of mosquitoes. Olfactory sensory neurons that express olfactory receptors (ORs) or ionotropic receptors (IRs) are housed inside hair-like sensilla that cover these olfactory appendages [5, 6]. A transduction cascade that sends a signal to the olfactory lobes in the cerebral ganglion of the insect is triggered when odorants bind to the ORs and IRs. Odorants interact directly with these receptors, and therefore differences in host preference between species could be reflected in differences in the expression or molecular structure of the receptors.

Each OR is a heteromeric ligand-gated ion channel that is encoded by the conserved co-receptor *Orco,* as well as a specific *Or* [7]. ORs differ in their tuning breadth with some ORs responding to a few odorants, whereas others respond to a wide variety of volatiles [8, 9]. IRs are also heteromeric ligand-gated ion channels, containing up to three different subunits that include one or two of the broadly expressed co-receptors *Ir25a, Ir76b* and *Ir8a* [10, 11].

In addition to the receptors, odorant binding proteins (OBPs) play an important role in olfaction, and like the receptors, also interact directly with odorants. OBPs are small, water-soluble carrier proteins that are highly expressed in the sensilla lymph. They transport hydrophobic odorants through the hemolymph to the receptors (reviewed in [12]). Currently, 63 putative *AgObp*s have been identified [13], but not all *AgObps* are expressed in female antennae [14]. Some OBPs almost certainly play a role in the transport of molecules outside the olfaction system, as some *AgObps* are expressed elsewhere in the body [13]. In addition, seven chemosensory proteins (CSPs), which are thought to have a similar function as OBPs, have been identified in *An. gambiae* s.l. [15].

The antennae express by far the largest repertoire of chemosensory genes in mosquitoes [16]. By contrast, the maxillary palps express a much smaller number of chemosensory genes [16, 17]. In mosquitoes, the palps are also responsible for CO_2_ detection, which in *An. gambiae* s.l. is modulated by three gustatory receptors; *AgGr22–24* [6]. Two *Ors* are highly expressed in mosquito palps; *AgOr8* and *AgOr28* in *An. gambiae* s.l. and their homologs in *Aedes aegypti* [17] and *Culex quinquefasciatus* [18]. In *An. gambiae* s.l. AgOR8 detects the non-human specific host odorant 1-octen-3-ol, whereas AgOR28 is broadly tuned [6]. Therefore, it is clear that the palps play an important role in mosquito host detection, but it is unknown if they could play a role in differentiating between host species’ odor profiles.

*An. gambiae* s.l. females are attracted to volatiles emanating from human sweat and breath, as well as CO_2_. Volatiles present on the human skin are believed to be responsible for the human-specific odor profile that attracts *An. gambiae* s.l. [19, 20]. Human sweat consists of over 350 volatiles, and several of these have been identified as mosquito attractants [21]. For example, *An. gambiae* s.l. females are attracted to a synergistic blend of ammonia, lactic acid and carboxylic acids [22, 23]. However, the kairomones that *An. gambiae s.l*. uses to differentiate between humans and alternative hosts remains unknown.

Olfaction gene expression divergence is known to be correlated to differences in host preference between closely related insect species. For example, the expression level of as many as 53% of the *Or*s and 55% of the *Obps* differs in the antennae of the generalists *D. melanogaster, D. simulans*, and their sibling-species *D. sechellia,* a specialist on the toxic *Morinda citrifolia*. This number is significantly more than expected based on other gene families [24]. Although neutral evolution may explain some of these changes, the expression level of several genes have undergone a major change along the *D. sechellia* branch, suggesting an association with the host shift [24]. For example, in *D. sechellia* the olfactory receptor *Or22a* is highly up-regulated*. Or22a* detects a compound emitted by the fruit of *D. sechellia’*s host plant *Morinda citrifolia* [25]. Additionally, *D. sechellia* lost six *Or* genes compared to its generalist sister-species *D. simulans,* which did not lose any [26]. Furthermore, accelerated olfactory receptor loss has also been associated with host specialization in *D. erecta* [27].

A link between differential olfaction gene expression and host preference has been observed in a mosquito species as well. A recent study of *Aedes aegypti* tied expression differences of *AeOr4* between the domestic *Ae. aegypti aegypti* and the sylvatic *Ae. aegypti formosus* to their differential host preference [28]. Furthermore, a previous comparison between the antennal transcriptome of *An. coluzzii* and *An. quadriannulatus* identified divergence in olfaction gene expression that could be related to their respective host preference [29]. This previous study was conducted during the light phase of the circadian cycle, which could have affected the results as it has been shown that olfactory gene expression can fluctuate across the circadian cycle [30]. Because *Anopheles* mosquitoes are night time feeders, we compared the transcriptome of the female antennae of this anthropophilic *An. colluzii* and zoophilic *An. quadriannulatus,* during the early dark cycle when both species are actively seeking hosts [31, 32]. In addition, we expand on the earlier work by comparing chemosensory gene expression between these species in the second olfactory organ, the maxillary palps.

## Results

### Host choice assay

We measured the preference of *An. coluzzii* and *An. quadriannulatus* laboratory strains to human odor vs cow odor in a dual choice olfactometer. Consistent with the host preference of these species in the field and previous studies, *An. coluzzii* significantly preferred human odor (77%, *N* = 770, *p* < 0.0001), Whereas *An. quadriannulatus* was significantly more attracted to cow odor (67%, *N* = 330, *p* = 0.0029). Therefore, the laboratory colonies of these species have largely preserved their natural host preference.

### Differential gene expression

Two replicate female antennae and two replicate maxillary palp transcriptome datasets were obtained for each of the species using RNAseq technology. For each library, between 30 and 46 million reads were generated (Table S1). After mapping of the reads to the *Anopheles gambiae* genome (Agam Pest Version 3.), we counted the uniquely mapped reads using the program HT-Seq [33], followed by statistical analyses using DeSeq2 package [34] on the R statistical platform. We only considered a gene to be expressed if more than 50 reads were mapped.

**Fig. 1:**
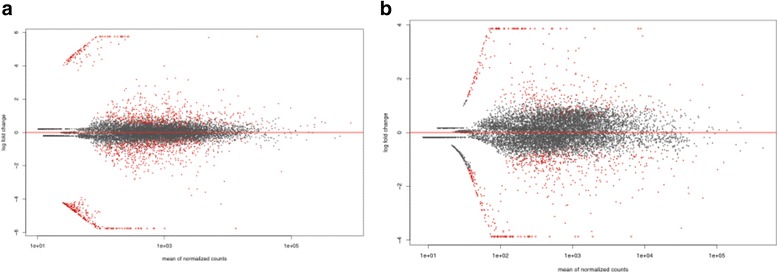
Plots of LogFC (Fold Change) against The Mean of Normalized Counts among the antennae (**a**) and maxillary palps (**b**) between *An. coluzzi* and *An. quadriannulatus*. Each dot represent a measure of abundance, with red colored dots indicating statistically significant differences in expression. logFC values above zero are those that are upregulated in *An. coluzzii* where as those below zero are upregulated in *An. quadriannulatus*

A total of 8857 and 9168 annotated genes were detected in the antennae of *An. coluzzii* and *An. quadriannulatus* respectively (Fig. 1a, Additional file 1). Of these, the expression of a total of 1595 genes was significantly diverged between the two species. The expression of a total of 708 genes was significantly enhanced in *An. coluzzii* antennae, whereas 887 genes were significantly up-regulated in *An. quadriannulatus*.

An average of 9207 and 8820 annotated genes were detected in the maxillary palps of *An. coluzzii* and *An. quadriannulatus* respectively (Fig. 1b, Additional file 1). Of these, 713 genes were differentially expressed between the two species, with 437 genes significantly enhanced in *An. coluzzii* and 296 in *An. quadriannulatus*.

### Olfactory receptors

**Fig. 2:**
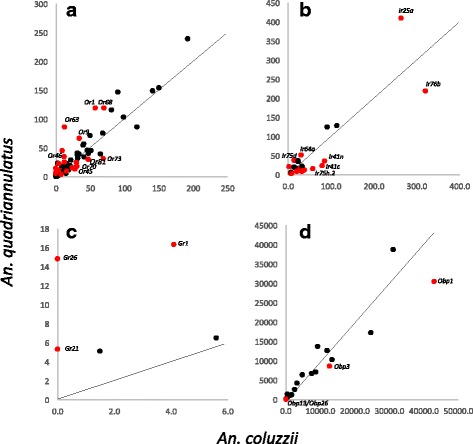
A comparison of the expression of four chemosensory gene families in the female antennae of the anthropophilic *An. coluzzii* and the zoophilic *An. quadriannulatus.* **a**: *Ors*, (**b)**: *Irs, (* **c)**: *Grs*, (**d)**: *Obps.* The line indicates equal expression between the two species. For the olfactory receptors *Orco* was excluded

**Fig. 3:**
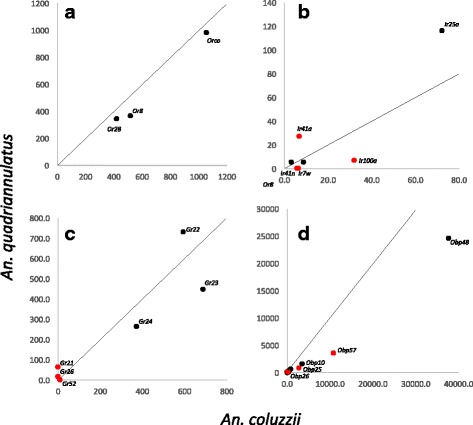
A comparison of the expression of four chemosensory gene families in the female maxillary palps of the anthropophilic *An. coluzzii* and the zoophilic *An. quadriannulatus.* **a**: *Ors*, (**b)**: *Irs, (* **c)**: *Grs*, (**d)**: *Obps.* The line indicates equal expression between the two species. For the olfactory receptors *Orco* was excluded

The expression of *Ors* was highly correlated between the two species in both the antennae (R^2^ = 0.99 including *Orco,* R^2^ = 0.84 excluding *Orco*) (Figure 2A), and the maxillary palps (R^2^ = 0.99 including or excluding *Orco*) (Fig. 3A). As expected, the co-receptor *Orco* was the most abundantly expressed *Or* in both olfactory organs, and was expressed at similar levels in both species. Of the remaining 74 known *Ors*, nine (*Or3, 4, 5, 20, 34, 37, 40, 52* and *58*) were detected (< 50 reads aligning) in the antennae or palps of neither species.

Although it is unknown what expression level of receptor genes signifies functional relevance, it is doubtful that genes detected at very low level play a significant functional role. Therefore, we focus here on receptor genes that are expressed at >5 rpkm. Of the remaining 66 *Ors,* nine were expressed above 5 rpkm and significantly up-regulated in *An. coluzzii* antennae (*Or36, 45, 57, 69, 70, 71, 72, 73, 81*) (Table 1, Additional file 1). Of these, *Or36, 45, 69, 70, 71, 73* and *81* stand out because they are enhanced between 1.6 and 2.4-fold in the anthropophilic *An. coluzzii.* All of these are expressed >14.6 rpkm in this species (Table 1)*.*

**Table 1:** Chemosensory genes significantly enhanced in the female antennae. Only receptor genes with rpkm > 5, or Obp/Csp genes with rpkm > 50 in either species are included. Fold change and log2 fold change values are presented as positive values if expression is enhanced in *An. coluzzii* and as negative values if expression is enhanced in *An. quadriannulatus*

Gene Stable ID	Gene name	RPKM colluzzi	RPKM quad	Fold Change	log2Fold Change	padj
AGAP009707	*Or71*	14.6	5.6	2.4	1.2	0.0301
AGAP009719	*Or73*	69.0	30.7	2.3	1.2	0.0000
AGAP003053	*Or45*	27.8	12.8	2.2	1.1	0.0002
AGAP004357	*Or57*	7.5	3.9	1.9	0.9	0.0259
AGAP009718	*Or70*	30.5	17.4	1.8	0.9	0.0000
AGAP001012	*Or36*	15.4	9.5	1.7	0.8	0.0087
AGAP013512	*Or81*	47.1	29.7	1.6	0.7	0.0083
AGAP009705	*Or69*	24.0	15.1	1.6	0.7	0.0326
AGAP009718	*Or72*	30.0	23.8	1.3	0.4	0.0253
AGAP004354	*Or26*	40.4	56.8	−1.3	−0.4	0.0407
AGAP002044	*Or77*	15.5	22.3	−1.4	−0.4	0.0321
AGAP007797	*Or23*	90.0	146.5	−1.5	−0.6	0.0081
AGAP009704	*Or68*	70.0	118.7	−1.6	−0.7	0.0000
AGAP008333	*Or9*	34.1	66.2	−1.8	−0.8	0.0081
AGAP009411	*Or21*	12.8	25.0	−1.8	−0.9	0.0012
AGAP009640	*Or1*	57.2	118.9	−1.9	−1.0	0.0000
AGAP009412	*Or50*	3.2	7.3	−2.1	−1.1	0.0000
AGAP009390	*Or53*	3.8	11.2	−2.5	−1.3	0.0022
AGAP009392	*Or46*	12.0	34.2	−2.6	−1.4	0.0000
AGAP011991	*Or61*	9.3	44.9	−3.4	−1.8	0.0028
AGAP005760	*Or33*	4.1	22.5	−5.0	−2.3	0.0000
AGAP011990	*Or64*	2.7	23.2	−5.8	−2.5	0.0000
AGAP011989	*Or63*	12.6	85.8	−6.1	−2.6	0.0000
AGAP009410	*Or18*	0.0	5.7	−32.6	−5.0	0.0000
AGAP009391	*Or30*	0.0	6.8	−49.4	−5.6	0.0000
AGAP009720	*Or74*	0.0	14.3	−75.0	−6.2	0.0000
AGAP007498	*Ir75k*	32.3	8.0	4.1	2.0	0.0000
AGAP001812	*Ir75h.2*	57.7	15.5	3.7	1.9	0.0000
AGAP012951	*Ir41c*	79.7	23.0	3.6	1.8	0.0000
AGAP013416	*Ir7w*	35.4	10.2	3.4	1.8	0.0000
AGAP000140	*Ir100a*	38.2	11.7	3.3	1.7	0.0000
AGAP013285	*Ir7u*	9.0	3.3	2.8	1.5	0.0000
AGAP013363	*Ir7i*	6.7	2.7	2.5	1.3	0.0000
AGAP002763	*Ir7t*	20.7	8.5	2.5	1.3	0.0000
AGAP003531	*Ir41n*	85.5	35.3	2.5	1.3	0.0000
AGAP013085	*Ir75g*	28.6	15.2	1.9	0.9	0.0272
AGAP011968	*Ir76b*	320.0	219.0	1.5	0.6	0.0283
AGAP010272	*Ir25a*	263.6	409.4	−1.5	−0.6	0.0000
AGAP004923	*Ir64a*	30.9	51.1	−1.6	−0.6	0.0013
AGAP004969	*Ir75d*	14.1	37.2	−2.4	−1.3	0.0000
AGAP001811	*Ir75h.1*	2.6	20.1	−6.7	−2.7	0.0000
AGAP004114	*Gr1*	4.1	16.3	−3.6	−1.8	0.0000
AGAP003260	*Gr21*	0.0	5.3	−43.9	−5.5	0.0000
AGAP006717	*Gr26*	0.0	14.8	−105.6	−6.7	0.0000
AGAP002905	*Obp13*	140.6	13.7	9.3	3.2	0.0000
AGAP012321	*Obp26*	150.8	57.4	2.4	1.3	0.0218
AGAP001409	*Obp3*	12,763.8	8566.4	1.6	0.6	0.0000
AGAP003309	*Obp1*	43,007.8	30,392.5	1.5	0.6	0.0375

Similarly, a total of 17 *Ors* were expressed above 5 rpkm and significantly up-regulated in *An. quadriannulatus* antennae (*Or1, 9, 18, 21, 23, 26, 30, 33, 46, 50, 53, 61, 63, 64, 68, 74* and *77*) (Table 1). Of these, all but *Or23, 26* and *77* are enhanced between 1.6 and 75.0 fold in this species, and all but *Or18, 30, 50,* and *53* are expressed >14.3 rpkm. *Or18, 30 and 74* are detected only in the antennae of this species, although at relatively low levels; between 5.7 and 14.3 rpkm.

Because some genes listed in Table 1 have relatively low fold change values (<2), the RPKM and fold change values for the replicate samples are reported in Additional file 2: Table S3. For all these *Ors,* the expression differences between species is consistent between replicate samples, although in the case of *Or69* the fold change for the second replicate of each species is considerably lower than for the first. For the majority of these *Ors,* including *Or69,* Rinker et al. [29] also reported considerable enhancement, although we should not necessarily expect their results to be the same because their sample represents day time expression.

As reported previously [6, 16], only two specific *Ors* are expressed at high level in the palps of *An. coluzzii*; *Or8* and *Or28.* This is true for *An. quadriannulatus* as well (Figure 3A, Table 2). No significant differences in the expression of these genes was observed. Several other *Ors* were detected at very low levels; < 2.8 rpkm in *An. coluzzii* and <5.0 rpkm in *An.quadriannulatus* (Additional file 1). However, as these correspond to the most highly expressed antennal *Ors,* their detection is likely due to a small amount of antennal contamination during dissection.

**Table 2:** Chemosensory genes significantly enhanced in the female maxillary palps. Only receptor genes with rpkm > 5, or Obp/Csp genes with rpkm > 50 in either species are included. Fold change and log2 fold change values are presented as positive values if expression is enhanced in *An. coluzzii* and as negative values if expression is enhanced in *An. quadriannulatus*

GENE stable ID	Gene name	RPKM coluzzi	RPKM quad	Fold change	Log2 Fold Change	padj
AGAP013416	*Ir7w*	6.4	0.0	27.7	4.8	0.0000
AGAP003531	*Ir41n*	5.8	0.0	19.0	4.2	0.0000
AGAP000140	*Ir100a*	31.9	6.7	3.8	1.9	0.0001
AGAP002904	*Ir41a*	6.9	27.1	−3.1	−1.6	0.0011
AGAP001173	*Gr52*	9.5	0.0	22.7	4.5	0.0000
AGAP006717	*Gr26*	0.0	16.1	−62.3	−6.0	0.0000
AGAP003260	*Gr21*	0.0	62.9	−198.7	−7.6	0.0000
AGAP012320	*Obp25*	2798.6	766.8	3.1	1.6	0.0010
AGAP011368	*Obp57*	10,799.9	3525.1	2.6	1.4	0.0141
AGAP012321	*Obp26*	240.5	74.4	2.5	1.3	0.0390
AGAP008054	*Sap3*	1117.4	2619.4	−2.3	−1.2	0.0000

### Ionotropic receptors

The expression of *Irs* was highly correlated between the two species if the co-receptors *Ir8a, Ir25a* and *Ir76b* were included (R^2^ = 0.801 for antennae*,* R^2^ = 0.842 for palps, Figure 2B). However, if the co-receptors were excluded, *Ir* expression diverged considerably, especially in the palps (R^2^ = 0.53 for antennae*,* R^2^ = 0.157 for palps, Figure 3B). Of the 44 annotated *Ir*s in the *Anopheles gambiae* genome, 11 were expressed (<50 reads) in the antennae of neither species (Additional file 1). The co-receptors, *Ir76b, Ir25a* and *Ir8a* are the most highly expressed *Irs* in both species: between 114.3 and 409.4 rpkm (Additional file 1). The use of these co-receptors appears to have diverged somewhat between the two species, with *Ir76b* significantly 1.5-fold enhanced in *An. coluzzii* versus *Ir25a* expressed 1.5-fold more in *An. quadriannulatus*. These differences were consistent within replicates (Additional file 2: Table S3).

Of the remaining *Irs*, ten (*Ir7i, 7t, 7u, 7w, 41c, 41n, 75g, 75h.2, 75k,* and *100a*) are significantly higher expressed in *An. coluzzii* and expressed >5 rpkm (Table 1). Of these, all but *Ir7u* and *7i* are expressed at >20 rpkm and between 1.9 and 4.1-fold enhanced in this species. The enhancement of *Ir75g*, the only *Ir* with fold change <2, was consistent between replicates (Additional file 2: Table S3).

In contrast, only three of the remaining *Irs* (*64a, 75d,* and *75h.1*) are expressed above 5 rpkm and significantly enhanced in *An. quadriannulatus*. All these are expressed >20 rpkm and are between 1.6 and 6.7-fold enhanced in *An. quadriannulatus* (Table 1). The enhancement of *Ir64a* the only *Ir* with fold change <2, was consistent between replicates (Additional file 2: Table S3).

A total of 14 *Irs* were detected in the maxillary palps of *An. coluzzii,* but only *Ir7w, 25a, 41a, 41n, 76b* and *100a* are expressed at >5 rpkm (Additional file 1)*.* As in the antennae, the co-receptor *Ir25a* was the most highly expressed ionotropic receptor in both species. Three *Irs* (*7w, 41n,* and *100a*)*,* are expressed above 5 rpkm and significantly enhanced in the maxillary palps of *An. coluzzii* (Table 2). Both *Ir7w* and *41n* were detected only in *An. coluzzii,* although at low level (6.4 and 5.8 rpkm respectively). *Ir100a* however is both the most highly expressed ionotropic receptor in *An. coluzzii* palps (31.9 rpkm), and is 3.8- fold enhanced in this species. This receptor is also significantly 3.3-fold enhanced in the antennae of this species, with a similar expression level (38.2 rpkm), but was barely detected in the body [16].

Only six *Irs* are expressed in *An. quadriannulatus* palps*,* five of which are detected at >5 rpkm. Of these, only *Ir41a* is significantly enhanced in the palp of *An. quadriannulatus*. It is also the most highly expressed specific *Ir* in the palps of this species (27.1 rpkm) and is 3.1-fold enhanced compared to *An. coluzzii*. This gene is not significantly enhanced in the antennae of this species, although expressed at a similar level (33.7 rpkm, 1.3 fold). Its mRNA is also mostly absent from the body, at least in *An. coluzzii* [16].

### Gustatory receptors

The expression of *Grs* showed little correlation between the two species in the antennae (R^2^ = 0.233, Figure 2C) (Fig. 2D). Of the 59 annotated *Grs* in the *Anopheles gambiae* genome, 39 were detected (<50 reads mapping) in the antennae of neither species, with the remaining *Grs* generally expressed <5 rpkm, (Additional file 1). In *An. coluzzii, Gr55* is the most highly expressed (5.6 rpkm). In *An. quadriannulatus, Gr1, Gr21* and *Gr26* are significantly enhanced and expressed >5 rpkm. Interestingly, *Gr26* is only detected in *An. quadriannulatus*, being expressed at 16.3 rpkm*.* (Table 1).

In contrast, nine *Grs* were detected in the palps of *An. coluzzii* and *An. quadriannulatus,* although these do not fully overlap (Additional file 3: Table S1, Figure 3c). *Gr22, 23* and *24,* which together encode the CO_2_ receptor, are of course by far the most highly expressed *Grs* in both species (262.9 < rpkm <729.8). Their expression level is similar between the two species, suggesting a similar sensitivity to CO_2_. The expression of *Grs* in the palps was therefore highly correlated between the two species (R^2^ = 0.901) if the CO_2_ receptor genes were included. However, this correlation disappeared completely if only the remaining *Grs* were examined (R^2^ = 0.001). A single *Gr, Gr52,* is specific to *An. coluzzii,* being detected only in this species, although in the lower range (9.5 rpkm, Table 2). None-the-less, its enhancement in *An. coluzzii* is highly significant (q < 0.0001). *Gr52* is expressed at low levels in the antennae of this species as well (3.3 rpkm), but not in *An. quadriannulatus.* Two *Grs* (*Gr21, 26*) are expressed >5 rpkm and significantly enhanced in the palps of *An. quadriannulatus*, while not detected in *An. coluzzii.* Of these, *Gr21* stands out by being both specific to *An. quadriannulatus,* and the most highly expressed *Gr* (62.9 rpkm) besides the CO_2_ receptor genes. *Gr26* is also specific to *An. quadriannulatus*, although with a lower expression level (16.1 rpkm). In the antennae both *Gr21* and *Gr26* are also expressed only in *An. quadriannulatus,* although at a much lower level (5.0 and 11.6 rpkm, respectively), suggesting a primarily palp-specific role.

### Odor-binding proteins

The expression of *Obps* was highly correlated between the two species (R^2^ = 0.915 for antennae*,* R^2^ = 0.990 for palps, Figure 2D and 3D). Thirty-four of the 63 annotated *Obp* *s* were detected in neither species in our antennae dataset (<50 reads), with another 11 expressed <50 rpkm in both species (Additional file 1). *Obps* comprise nine of the 10 most highly expressed genes in the antennae. Because of their high overall expression level relative to the receptor genes, we focus here only on those with expression >50 rpkm. Four *Obps* (*Obp1, 3, 13,* and *26*) are significantly enhanced in *An. coluzzii* antennae and expressed >50 rpkm (Table 1). However, of these only *Obp1* and *3* are highly expressed with rpkm values of 43,008 and 12,764 respectively. These two genes are 1.5 to 1.6-fold enhanced in *An. coluzzii,* with consistent expression differences between species across replicates (Additional file 2: Table S3). The expression level of *Obp13* and *26* is below 151 rpkm, which is in the very low range for *Obps*. No *Obp* expressed >50 rpkm was enhanced in *An. quadriannulatus* antennae*.*

Despite the high correlation between *Obp* expression in the maxillary palps of the two species, the expression of several *Obps* has diverged substantially. Four *Obps* expressed >50 rpkm (*Obp25, 26, 57* and *67*) are significantly enhanced in *Anopheles coluzzii*. Of these, *Obp25* and *Obp57* stand out. They are among the four most highly expressed *Obps* in the maxillary palps of *An. coluzzii* (2798 and 10,800 rpkm respectively), and are considerably enhanced (3.1 and 2.6-fold respectively). Both *Obp25* and *Obp57* are expressed at much lower level in the antennae (407.1 and 63.0 rpkm, respectively) and therefore appear to be mostly palp-specific. In contrast, no *Obp* expressed at >50 rpkm is significantly enhanced in *An. quadriannulatus* palps. Interestingly, overall *Obp* expression in the *An. quadriannulatus* palps is 1.8-fold lower compared to *An. coluzzii* (33,148 vs 58,482 rpkm). This is not the case for *Obp* expression in the antennae.

### Chemosensory proteins

Like the *Obps*, *Csp* genes probably function as odorant carrier molecules and are expressed at levels considerably higher than the receptors (as high as 4992 rpkm in the antennae and 15,504 rpkm in the maxillary palps, Additional file 1). In both the palps and antennae, the expression of these genes was highly correlated between species (R^2^ = 0.995 and R^2^ = 0.977, respectively). All seven annotated *Csp* genes, following Iovinella et al. [15], were detected in the antennae of both species*,* although several at very low level*.* No *Csp* showed enhanced expression in the antennae of either species. All seven *Csps* were detected in the palps of both species as well, with six expressed at >50 rpkm. One of these*, Sap3*, is significantly enhanced (2.3-fold) in the palp of *An. quadriannulatus* (Table 2). This gene is expressed at more similar levels (1.4 fold) in the antennae of the two species. The *Sap3* gene is the second most highly expressed *Csp* in the palp of both species.

Overall *Csp* expression is approximately 3-fold higher in the palps of *An. coluzzii* than its antennae, and 2-fold higher in the palps vs antennae of *An. quadriannulatus.* Interestingly, *Sap1* is the 3rd and 6th most highly expressed gene overall in the palps of *An. coluzzii* and *An. quadriannulatus* (15,404 and 11,762.6 rpkm, respectively), whereas it is expressed at much lower levels in the antennae (658 and 655 rpkm respectively). Vice versa, *Csp3* appears to be largely antennae specific (3719 and 4992 rpkm in *An. coluzzii* and *An. quadriannulatus* respectively), with very low expression in the palps (92,7 and 64,7 rpkm in *An. coluzzii* and *An. quadriannulatus* respectively).

### Gene ontology analysis

A gene ontology analysis (GO) was conducted using the online gene ontology database pantherdb (www.pantherdb.org) to recover descriptions of molecular function. We performed the GO analyses only on genes that were upregulated in the antennae or the maxillary palps of each species. This resulted in 708 antennal genes in *An. coluzzii* and 887 antennal genes in *An. quadriannulatus*. For the maxillary palps, 437 and 296 genes met these criteria in the two species, respectively. Not surprisingly, the molecular functions of the significantly enhanced genes are connected to olfaction (e.g., “protein binding”, “receptor activity”) and signal transduction (e.g “transporter activity”, “transmembrane transporter activity”). The Panther GO Biological Process classification returned “Sensory perception of smell” and “Chromatin Assembly” as the top two terms. Furthermore, we tested for enrichment of specific protein families, which showed that, based on number of genes hit against protein class, the top three protein families enriched in *An. coluzzii* antennae were Hydrolase (PC00121), Receptor (PC00192), and Nucleic Acid Binding (PC00171). In contrast, based on genes upregulated in *An. quadriannulatus* antennae showed that the protein families enriched were Oxidoreductase (PC00176), Hydrolase (PC00121), and Transferase (PC00220).

In the maxillary palps, the enriched protein classes were as follows. In *An. coluzzii*, they were Oxidoreductase (PC00176), Hydrolase (PC00121) and Nucleic Acid Binding (PC00171). In *An. quadriannulatus*, the top three protein families were Hydrolase (PC00121), Transporter (PC00227), and Oxidoreductase (PC00176). The entire list of protein families for each tissue is shown in Additional file 4: Table S4.

## Discussion

The host seeking behavior of the sister species *An. gambiae* and *An. coluzzii* is primarily modulated by their olfactory system [1]. It is therefore expected that their preference for human hosts has a strong basis in the genes that make up the most important components of this system. The olfactory system is primarily housed in the antennae, which express a large number of olfactory genes. However, the maxillary palps also play an important role in host detection as they contain the receptor for CO_2_, as well as two olfactory receptors that detect the host kairomone 1-octen-3-ol and a range of other chemicals [7]. Additionally, olfactory receptors are expressed in the labellum of mosquito proboscises which may play a role in discrimination host at short range [35, 36].

Here we compared the transcriptomes of the antennae and maxillary palps of two species of malaria mosquitoes with diverging host preference during the dark cycle when they are actively seeking hosts: the anthropophilic *An. coluzzii* and the zoophilic *An. quadriannulatus*. We focused on five chemosensory gene families that interact directly with odorants. The expression profiles of these chemosensory gene families generally show a high correlation between the two species, but considerable differences in expression of individual chemosensory genes are present. These could point to genes responsible for the diverging host preference between these closely related species.

Divergence in olfaction gene expression associated with host specialization has been observed in other insects. Recently, it was shown that divergent expression of *AaOr4,* an olfactory receptor without an ortholog in *An. gambiae s.l.,* between an anthropophilic and zoophilic form of *Aedes aegypti* contributes to differences in host preference [28]. Similarly, specialization to host plants has been accompanied by differential expression of olfactory receptors between closely related *Drosophila* species. *Or* expression in the antennae differs markedly between the generalists *D. melanogaster, D. simulans*, and their specialist sister-species *D. sechellia*, with the expression of up to 53% of *Or*s differing between species pairs [24]. Therefore, the enhanced expression of some of the chemosensory genes in *An. coluzzii* could very well be linked to their preference for human odor.

In the antennal transcriptome of *An. coluzzii,* seven *Ors* (*Or36, 45, 69, 70, 71, 73* and *81*) and eight *Irs* (*Ir7w, 7t, 75h.2, 75g, 75k 41n, 41c,* and *100a*) stand out by being among the more highly expressed receptor genes, while also being considerably up-regulated compared to *An. quadriannulatus* (1.6 to 4.8-fold)*.* Previous studies have reported the response of 56 AgORs to a range of odorants [8, 9]. Of the seven *Ors* enhanced in *An. coluzzii* that are highlighted here, only OR36 showed a positive response to the tested odorants. In comparison with other tested ORs it appears to be narrowly tuned, and it responded strongly to only two odorants, neither of which are of human origin [9]. However, 346 volatiles have been identified in human sweat [21], and only a subset of these volatiles have been tested*.*

The relative role of ORs vs IRs in host differentiation is not entirely clear. IRs are thought to detect several important human kairomones; lactic acid, carboxylic acids and amonia [37–40]. Although *IRs* play a role in the detection of host odors, this role may depend on the presence of CO_2_, as *Aedes aegypti* females in which *Orco* is knocked-out are attracted to human hosts, but can no longer distinguish between different vertebrate hosts [41]. This suggests that in *Ae. aegypti* IRs do not play a role in host differentiation. However, it is not clear that this rules out a potential role of IRs in human host preference in *Anopheles.* The divergence in *Ir* expression in the olfactory tissues observed in this study suggests some species-specific role of these receptors.

OBPs carry odorants through the hemolymph to olfactory receptor neurons and are essential for the proper function of the olfactory system [12]. For example, *Drosophila melanogaster* that carry a mutant OBP called LUSH, do not detect an aggregation pheromone [42], and *Obp57d* and *Obp57e* are linked to diverging oviposition behavior between *Drosophila* species [43]. In *An. gambiae* RNAi knock-down of *AgObp1* abolishes the antennal response to indole [44]. Interestingly, knocking down *AgObp4,* which is expressed primarily in the antennae, also suppresses blood feeding from a membrane feeder which does not rely on odor cues [45]. In *Culex quinquefasciatus* knockdown of *CqObp1* results in lowered response to an oviposition pheromone [46]. The importance of some OBPs to the olfactory system of insects is therefore well established, but it is not clear if they contribute to host selectivity [12, 17].

We note the divergence in the expression of several *AgObps* between the two species. In the antennae, the highly expressed *Obp1* and *3* are enhanced 1.5 to 1.6 -fold in *An. coluzzii*. More remarkable however is the enhancement of the highly expressed *Obp25* and *57* in the palps of this species. These two *Obps* are enhanced 2.6 to 3.1–fold in this species compared to *An. quadriannulatus*. In addition, *Obp25* is expressed in the antennae of *An. coluzzii* at only appr. 0.15-fold level compared to the palps, and *Obp57* is expressed at very low level in the antennae (63.0 rpkm). Additionally, both these *Obps* are detected only at very low levels in the body of *An. coluzzii* [16]. This suggests a mostly palp-specific function of these genes.

Although the maxillary palps obviously have an important role in host seeking by detecting CO_2_ [47], to our knowledge no one has examined if these organs function in host differentiation in mosquitoes. The small and similar *AgOr* repertoire in the palps suggests that they do not [6]. However, the considerable difference in the expression of several palp-specific *Obps,* as well as *Ir100a* and *Ir41a,* does suggest a species-specific role of the maxillary palps in olfaction.

In addition, several *Grs* showed a species-specific expression pattern in the maxillary palps. *Gr52* is specific to *An. coluzzii,* and *Gr21* and *Gr26* are both specific to *An. quadriannulatus* palps in this study, although *Gr21* was detected at low levels in *An. coluzzii* palps in a previous study [16]. We found an approximately 3-fold higher expression of *Gr52* in the palps vs the antennae in this species. However, a previous study found that the transcript of this gene is present in the whole bodies, antennae and palps of this species at similar levels, with only approximately 1.5-fold enhancement in the palps [16]. Therefore, this gene likely has a function other than a gustatory or olfactory one.

One the *An. quadriannulatus* specific *Grs*, *AgGr21,* is homologous to *DmGr64a* and *DmGr61a* [48]*,* which function as sugar receptors in *Drosophila melanogaster* [49, 50]. Interestingly, it was recently found that several sugar receptors are expressed in olfactory neurons in *Drosophila* antennae and/or palps, and one of these is co-expressed with *Orco.* This could suggest a hitherto unknown role of these receptors in olfaction [51]. The other *An. quadriannulatus* specific *Gr, Gr26,* has a relatively low expression level and it is unknown if this level of expression for a receptor gene (approx. 12 rpkm) could indicate a species-specific role in olfaction. Neither *AgGr21* nor *AgGr26* has a known ortholog in *Aedes* or *Drosophila.*

Although the *An. quadriannulatus* strain examined in this study preferred bovine odor, this was not the case in a previous study in which this species did not distinguish between human and cow odor [3]. Furthermore, in an additional study it blood fed equally on a human and equal sized calf [52]. This suggests that *An. quadriannulatus* is more of a generalist than *An. gambiae* with a wider host preference*.* The number of expressed and enhanced olfaction genes in *An. quadriannulatus* indicates it likely also responds to a complex blend of odors during host seeking, rather than focusing on a few universal mammalian kairomones.

Rinker et al. [29] previously compared the daytime transcriptomes of the antennae of *An. coluzzii* and *An. quadriannulatus.* When comparing that study with our results, we observe considerable agreement. Specifically, when looking at the antennal *Ors, Irs* and *Obps* highlighted in our study, Rinker et al. [29] also found most of these genes to be enhanced in either *An. coluzzii* (*Or36, Or45, Or69, Or70, Or73, Or81, Ir7w, Ir7u, Ir75g, Ir75h.2, Ir41n, Ir41c, Ir100a, Obp1* and *Obp3*) or *An. quadriannulatus* (*Or1, Or9, Or33, Or46, Or50, Or63, Or64, Or68, Ir75d* and *75h.1*) [29] (Additional file 4: Table S4 and Additional file 5: Table S5). The expression differences of these genes can therefore be interpreted as representing real consistent biological differences between the species. Differences observed between the two studies could be due to fluctuations of gene expression during the circadian cycle, differences in rearing conditions, or genetic drift in laboratory colonies.

Our study used a laboratory strain of *An. coluzzii.* Until recently this species was considered the *An. gambiae* M form, but was elevated to species level on the basis of ecological differences in larval breeding, premating isolation, and several small highly differentiated genomic regions [53] that encode for premating isolation between it and the *An. gambiae* S form, now known as *An. gambiae* s.s. [54]. Both species are highly anthropophilic and have very limited genomic divergence. Therefore, their human host preference is almost certainly an ancestral trait with a shared genetic basis*.* We therefore expect that the results from our study will be largely applicable to *An. gambiae* s.s*.* as well.

The olfactory system of *Anopheles* mosquitoes plays an important role in other aspects of their biology. It is used to locate sugar sources such as nectar, as well as oviposition sites. We do not know from what source or how often or *An. quadriannulatus* females obtain sugar meals. However, it has been established that *An. coluzzii* stops responding to honey volatiles five days after emergence, and starts being strongly attracted to human odor at that point [55]. Observations of *An. quadriannulatus* strains kept in our laboratory indicates that they also switch to host seeking around this time. The two species appear to have a similar larval ecology, as larvae of both species can be found in shallow, open, sunlit fresh water pools [56, 57]. In any case the search for oviposition-sites does not begin until 48 h after blood feeding. None-the-less, we cannot rule out that the divergence in chemosensory gene expression observed in this study are due other biological differences between the two species than host preference.

## Conclusion

This comparison of gene expression in the olfactory organs of the anthropophilic *An. coluzzii* and the zoophilic *An. quadriannulatus* identified chemosensory genes that may underlie differences in host preference between the two species. Several medium high expressed olfactory and ionotropic receptors show considerable enhancement in the antennae of either species. In addition, expression of several *Obps* in the antennae and maxillary palps is considerably diverged between the two species. Finally, a small number of gustatory receptors in the maxillary palps were found to be species-specific. It is likely that some of the chemosensory genes identified here play a role in the differential host preference between the two species.

## Methods

### Mosquito rearing

Study populations in this study were lab colonies of *An. colluzzii* and *An. quadriannulatus*. The laboratory strains of *An. coluzzii* (formerly classified as *An. gambiae* M form*,* GASUA) originate from Suakoko, Liberia, and the *An. quadriannulatus* (SANQUA) colony originates from female mosquitoes collected in Sangwe, Zimbabwe, and reared in the insectaries at Wageningen University, The Netherlands (host choice experiment), and Texas A&M University, College Station, TX, USA (RNAseq analyses). Colonies were maintained at 25 °C, 75–85% relative humidity and a light:dark photoperiod of 12 h. Female mosquitoes were blood fed on defibrinated rabbit blood using an artificial membrane feeding system. Larvae were fed finely ground fish food (Tetramin, Melle, Germany), and were maintained at densities of approximately 150 per 2 L container. Pupae were collected and placed into cages at densities of two cups of 150 pupae per cage.

To obtain mosquitoes for RNAseq analyses newly emerged mosquitoes were removed daily from rearing cages. To obtain mosquitoes from the same age, pupae that failed to eclose were transferred to new cages. Male and female mosquitoes were kept in the same cage and maintained on a 5–10% sucrose solution until tissue dissections. Therefore, females were allowed to mate, but not to blood-feed. Insemination rate was found to be high (82%) in six-day old females. Species identity of the mosquito colonies was confirmed using the RFLP-PCR develop by Fanello et al. [58].

### Dual odor-choice assay

A total of 750 female *An. colluzzii* and 330 *An. quadriannulatus* females were used to determine their preference for human or cow odor. Seventy-five to eighty mosquitoes were placed in release cages the night before experiments with access to a wet cotton ball for hydration. The next morning the release cage was attached to the dual-choice olfactometer. The human odor sample consisted of a socks worn by volunteers for 24 h, and the cow odor sample consisted of a panty hose that had been tied around the leg of a cow for 24 h. Odor sources were switched between the left and right port of the olfactometer between runs. A single CO_2_ source, placed between the two odor ports was used as activator. Conditions during the experiments were as follows: temperature = 26–28 °C, humidity = 55–75% inside olfactometer, 80% in front of port holes, air-speed 018–0.22 ms^−1^, and released [CO_2_] = 4.5%. Mosquitoes were released into the olfactometer during the dark-cycle for 15 min. Experiments were conducted under semi-dark conditions. Mosquitoes that remained in the wind tunnel and did not enter an odor port during the experiment were discarded.

### Olfactory tissue dissections

Shortly after dark, anophelines begin their host seeking activity following their normal circadian patterns, and *Orco* expression in *An. coluzzii* peaks at this time. Hence, to capture the olfactory gene expression in these mosquitoes during their host-seeking phase, we collected their antennae and maxillary palps soon after dark. First, we immobilized female mosquitoes shortly after the start of the dark cycle by placing them at −20 °C for about a minute, and then placed them inside petri-plates on ice. The antennae and maxillary palps were each separately removed from immobilized mosquitoes while placed under a 10× dissecting microscope, and immediately stored in RNAlater***®*** Ice (Ambion). Between 300 and 500 six-day old females were dissected for each replicate and two replicates per species were included for a total of four samples per species. Samples were stored at −20 °C until RNA extraction, which was carried out for all samples within 48 h after dissection.

Total RNA was isolated from each sample using miRNeasy (Qiagen) columns using the protocol provided by Qiagen. A Qubit fluorometer (Life Technologies) was used to quantify RNA originaly. Subsequently, the RNA was quantified using a NanoDrop spectrophotometer (Thermo Scientific) followed by a quality assessment with a RNA Pico LabChip analysis on an Agilent BioAnalyzer 2100 (Agilent Technologies) by the Agrilife Genomics Center at Texas A&M University. Messenger RNA was enriched from about 1 μg of total RNA using the Poly-A enrichment method and cDNA libraries were prepared using an Illumina TruSeq RNA Library kit (Illumina). All libraries were sequenced over two lanes of Illumina HiSeq 2500 platform running in high-output mode, and generating 100 bp single-end reads. Preparation and sequencing of libraries were both performed at the Agrilife Genomics core facility at Texas A&M University, College Station, Texas. Approximately 30–46 million reads with an average read of 100 bp were generated for each replicate sample and used for further analysis.

### RNA sequencing analyses

Two replicate female antennae and two replicate maxillary palp transcriptome datasets were obtained for each of the two species using RNAseq technology on the Illumina HiSeq 2500 platform***.*** For each library, between 20 M and 30 M reads were generated. The quality of raw data was first assessed using FastQC (version 0.10.0). Fastq files were trimmed and QC-filtered using NGSQCTooklit package [59], and only the reads with at least 80% of the bases at phred score = > 30 were retained, and the rest were discarded. After quality filtering and trimming, an average of 96% of the reads were retained in each library. Filtered-trimmed files were then aligned to the mtGenome from *An. gambiae* assembly Pest Version 4.2 (agamp4) using the short-read aligner Bowtie2 [60] to remove mitochondrial RNA and/or rRNA contamination that was not removed during library preparation. We found 6–10% of the reads were mapping to mitochondrial rRNA for each of the samples, and we subsequently excluded the reads mapping to the mitochondrial genome for differential gene expression analyses. Reads that did not map to mtDNA reference were extracted out into a new Fastq file. These reads were aligned to *Anopheles gambiae* reference genome (PEST strain, Assembly version 4.2, retrieved on May 13th 2015 from **www.vectorbase.org**), using the de novo splice assembler STAR [61, 62]. After mapping, the reads mapping to ‘exon’ features were counted using the R package HTSeq (geneset: AgamP4.2, retrieved from **www.vectorbase.org** on May 13th 2015). Aligned reads were counted using the tool HTSeq-Count [33]. Reads that mapped only once in the genome were used for the differential expression analyses. Characteristics of data used for analysis are provided in Table S1. Reads mapping to multiple loci were excluded from the analyses. The counts data for each annotated feature on the reference genome was used for downstream statistical comparisons.

Clustering of the variance-stabilized transformed counts indicated that there was relatively little variation between biological replicates compared to between species for both antennae and maxillary palp samples. It was therefore deemed to use them as replicates in statistical analysis.

### Analysis of differential gene expression

With the ever-increasing variety of software packages to estimate differential expression, and the absence of standardized RNAseq specific methods [63], the repeatability, and the interpretation of the biological significance of expressed genes will rest on the variance structure unique to each dataset, and the statistical methods. To this end, we decided to use the popular and well-reviewed statistical [64] analysis package for DGE analysis - namely DESeq2 [34]. The DESeq2 statistical package is available as part of the Bioconductor suite on the R platform, and uses implementations of negative binomial modeling of counts data.

In DESeq2, size factors for each dataset were calculated to normalize library sizes across replicates, and overall means and variances were determined using a negative binomial distribution model. Genes were considered to be differentially expressed if q < 0.05 after correcting for multiple testing. Differentially expressed genes were tested by the GLM likelihood ratio test (after fitting negative binomial models and estimating dispersion), and considered significantly differentially expressed if the adjusted *P*-value, (padj) <0.05. Reported fold change values reported were calculated using the 2^^(Log2FoldChange)^ equation in excel on the Log2 fold changes obtained from the DESeq analysis.

### Gene ontology analysis

Genes that met the following criteria were used for gene ontology analysis. Genes that appeared on the consensus list by virtue of having FDR < 0.05 under the edgeR analysis and padj < 0.05 in the DESeq analysis between antennae or maxillary palps of the two species. GO Annotation was performed using the gene ontology analysis engine Pantherdb (www.pantherdb.org). The gene ids were retrieved from Ensembl release 22 via Biomart (http://metazoa.ensembl.org/index.html). GO annotation was used for assessment of the molecular function of genes that were differentially expressed between species. GO annotation associates analyzed transcripts with terms from hierarchical vocabularies describing, e.g., molecular function or biological process.

## Additional files

Additional file 1: Gene expression data for the antennae and maxillary palps of female Anopheles coluzzii and Anopheles quadriannulatus. (XLSX 3020 kb)

Additional file 2: Table S3. RPKM and fold change values for replicate samples for chemosensory genes with small (fold change <2), yet significantly different expression between species. (XLSX 45 kb)

Additional file 3: Table S1. Mapping statistics. (XLSX 27 kb)

Additional file 4: Table S4. Protein families enriched in An. coluzzii and An. quadriannulatus antennae and maxillary palps. (XLSX 33 kb)

Additional file 5: Table S5. Gene expression data for chemosensory genes in antennae reported in this paper and in Rinker et al. [29]. RPKM and fold change values between replicate antennae samples for genes listed in **Table 1.** with a Fold Change values <2. Also included are the expression levels for these genes reported in Rinker et al. [29]. (XLSX 80 kb)

## Abbreviations

*An*: *Anopheles*
*Csp*: Chemosensory receptor
*D*: *Drosophila*
GO: Gene ontology
*Gr*: Gustatory receptor
*Ir*: Ionotropic repector
*Obp*: Odorant binding protein
*Or*: Olfactory receptor
